# MiR-155 deficiency and hypoxia results in metabolism switch in the leukemic B-cells

**DOI:** 10.1186/s12935-024-03437-8

**Published:** 2024-07-18

**Authors:** Elena Golovina, Tomas Heizer, Lenka Daumova, Martin Bajecny, Simona Fontana, Valentina Griggio, Rebecca Jones, Marta Coscia, Chiara Riganti, Karina Savvulidi Vargova

**Affiliations:** 1 Institute of Pathological Physiology, First Faculty of Medicine, Charles University, Prague, Czech Republic; 2 Institute Biocev, First Faculty of Medicine, Charles University, Vestec, Czech Republic; 3 Center for Advanced Preclinical Imaging (CAPI), First Faculty of Medicine, Charles University, Prague, Czech Republic; 4 Oncological Pharmacology Laboratory, Department of Oncology, University of Torino, Torino, Italy; 5 Department of Molecular Biotechnology and Health Sciences, University of Torino and Division of Hematology, A.O.U. Città Della Salute E Della Scienza Di Torino, Torino, Italy

**Keywords:** B-cells, Hypoxia, miRNA, CRISPR/Cas9, Leukemia, Gene expression, Cell viability

## Abstract

Hypoxia represents one of the key factors that stimulates the growth of leukemic cells in their niche. Leukemic cells in hypoxic conditions are forced to reprogram their original transcriptome, miRNome, and metabolome. How the coupling of microRNAs (miRNAs)/mRNAs helps to maintain or progress the leukemic status is still not fully described. MiRNAs regulate practically all biological processes within cells and play a crucial role in the development/progression of leukemia. In the present study, we aimed to uncover the impact of hsa-miR-155-5p (miR-155, *MIR155HG*) on the metabolism, proliferation, and mRNA/miRNA network of human chronic lymphocytic leukemia cells (CLL) in hypoxic conditions. As a model of CLL, we used the human MEC-1 cell line where we deleted mature miR-155 with CRISPR/Cas9. We determined that miR-155 deficiency in leukemic MEC-1 cells results in lower proliferation even in hypoxic conditions in comparison to MEC-1 control cells. Additionally, in MEC-1 miR-155 deficient cells we observed decreased number of populations of cells in S phase. The miR-155 deficiency under hypoxic conditions was accompanied by an increased apoptosis. We detected a stimulatory effect of miR-155 deficiency and hypoxia at the level of gene expression, seen in significant overexpression of *EGLN1*, *GLUT1*, *GLUT3* in MEC-1 miR-155 deficient cells. MiR-155 deficiency and hypoxia resulted in increase of glucose and lactate uptake. Pyruvate, ETC and ATP were reduced. To conclude, miR-155 deficiency and hypoxia affects glucose and lactate metabolism by stimulating the expression of glucose transporters as *GLUT1, GLUT3*, and *EGLN1* [Hypoxia-inducible factor prolyl hydroxylase 2 (HIF-PH2)] genes in the MEC-1 cells.

**Supplementary Information:**

The online version contains supplementary material available at 10.1186/s12935-024-03437-8.

## Introduction

Leukemic cells, due to their high-energy demand, are forced to balance the oxygen deprivation in their original microenvironment by altering their metabolism [1]. One of the main manifestations of hypoxia at the cellular level is the modulation of cell proliferation. In general, cells adapt to hypoxia by expression of hypoxia-inducible factor (HIF) proteins. The HIF family is highly sensitive to intra-tissue pO_2_ and regulates more than 200 genes which reestablishes oxygen homeostasis and promotes cell survival in hypoxia [2]. The key molecule of this pathway is Hypoxia Induced Factor 1α (*HIF-1α*) which is often upregulated in tumors due to intratumoral hypoxia or by activation of some oncogenic pathways such as PI3K, TGFβ, MYC, and NOTCH. *HIF-1α* is overexpressed in the early stages of hypoxia (few hours) and is associated with aggressive behavior of leukemic cells and with low survival of patients [3]. Besides *HIF-1α,* the hypoxia-related genes (*EGLN1, VHL, HK1, HK2, LDHA, VEGFA, PTEN, PIK3CA*) participate in the modulation of cell proliferation and metabolism and thus maintain the leukemic status [3]. CLL cells show high plasticity to hypoxic conditions that results in metabolic adaptation, mainly involving glucose and pyruvate [4].

In hypoxia, malignant cells increase their proliferation and glucose uptake through glycolysis [5]. The key sensors of glycolysis during hypoxic conditions in leukemic cells are glucose transporters (especially *GLUT1* and *GLUT3*) [6]. In humans, there exists three families of genes that code glucose transporters: (1) *SLC2A* genes that code sodium-independent glucose transporters (facilitated transport, GLUT proteins); (2) *SLC5A* genes, that code sodium-dependent glucose symporters (secondary active transport, SGLT proteins), and (3) *SLC50A* genes, which code SWEET protein [7]. The family of sodium-independent glucose transporters contains 14 members *GLUT1-GLUT14*, encoded by *SLC2A1-SLC2A*14 genes, respectively [8]. Measurement of the level of glucose transporters (glucose uptake) is used as a diagnostic technique for cancers (18F-deoxy-glucose positron emission tomography, FDG-PET) [9]. Therefore, therapeutic inhibition of glucose transporters may be an approach for future treatments for patients with different malignancies. Small non-coding microRNAs (miRNAs) could serve as mRNA targeted inhibitors (small therapeutic inhibitory molecules). It was described that miRNA-195-5p inhibits *GLUT3* resulting in the inhibition of glucose uptake and growth of bladder cancer cells [10]. Similarly, inhibition of *GLUT1* by miRNA-125a-5p was described in thyroid carcinoma [11]. There is evidence that miRNAs are important regulators of glucose metabolism [12]. Interestingly, there are only few papers describing the direct effect of miR-155 on glucose metabolism but none in the relation of miR-155 to CLL cells and hypoxia. For example, Kim et al., 2018 found that the deletion of miR-155 in breast cancer cells abolishes the glucose uptake [13].

Hypoxic cells show high levels of lactate which serves as a signaling molecule, as an end product of anaerobic glycolysis, and is involved in fatty acid synthesis and redox homeostasis [14]. Lactate dehydrogenase (LDHA) catalyzes conversion of pyruvate into lactate. Future miRNA-based therapies could be used to modulate tumor/leukemic cell metabolism [15].

HypoxamiRNA (hypoxia related miRNA) significantly modulates gene expression through its targets thus improving adaptation of the leukemic cells to hypoxia [16]. The most studied hypoxamiRNAs is miR-210 which is significantly overexpressed in several cancers during hypoxic conditions. Overexpression of miR-210 is associated with a poor prognosis [17]. Here we focus on the oncomiRNA and possible hypoxamiRNA, miR-155 in CLL (MEC-1 cell line). MiR-155 is an essential regulatory molecule involved in multiple physiological processes, including hematopoietic lineage differentiation, immune response, and inflammation [18, 19]. Moreover, expression of miR-155 is directly associated with cancer progression and disease aggressiveness, including in CLL [20–22]. MiR-155 is expressed ubiquitously in different cells thus any knowledge about its function will be valuable in every research area.

Hypoxia related genes as *HIF1α* and *VHL* are direct validated targets of miR-155 [37]. MiR-155 acts as an inhibitor of tumor-suppressor pVHL that controls degradation of HIF1α protein. Overexpression of miR-155 leads to HIF1α protein stability in primary CLL cells [23]. Griggio et al., 2020 reported an aberration in the tumor-suppressor gene *TP53* that is also associated with higher expression level of *HIF1α* [24] which has an unfavorable prognosis in CLL patients [25].

In this study, we tracked the role of oncogenic and possible hypoxic related miR-155 in MEC-1 cells. We aimed to answer the question whether miR-155 deficiency affects cell proliferation, cell cycle, and the gene expression profile under hypoxic versus normoxic conditions. We found that hypoxia leads to the modulation of miRNA/mRNA network and in the absence of miR-155 in MEC-1 cells impair cell proliferation, switches glucose and lactate metabolism.

## Materials and methods

### Cell line and experimental model

We acquired the original MEC-1 cell line (#ACC 497, DSMZ) as a kind gift from Prof. Marek Mraz, M.D., Ph.D. (Laboratory of Microenvironment of Immune Cells, Central European Institute of Technology (CEITEC MU) and University Hospital Brno, Czech Republic). We modified the original MEC-1 cells using CRISPR/Cas9. A CRISPR/Cas9 plasmid (U6gRNA-Cas9-2A-GFP MiR155) was introduced into MEC-1 cells by nucleofection by Amaxa Nucleofector II (Lonza) with use of the B-cell nucleofection kit (#VPA1001, Lonza) and U-015 program. The CRISPR/Cas9 plasmid we ordered from Sigma-Aldrich, was designed at our laboratory and targeted the mature hsa-miR-155-5p sequence. More information about the creation and validation of miR-155 deficient MEC-1 clone #48 are in the Supplemental Fig. 1. MEC-1 cell line was cultured in IMDM medium (#LM-I1090, Biosera) supplemented with 10% of fetal bovine serum (#FB-1090, Biosera), 1% of P/S (#P4333, Sigma Aldrich) at 37 °C in 5% CO_2_ incubator. We followed the recommended protocol for cell culturing from DSMZ for MEC-1 cells (#ACC-497). The hypoxic MEC-1 cell line was incubated in hypoxic glove box (Coy O_2_ Controlled InVitro Glove-Box–Hypoxia Chamber, Genetica) in IMDM medium supplemented with 10% of fetal bovine serum, 1% of P/S at 37 °C, 1% O_2_ (for optimizing experiments we used 0.2%, 1% and 5% O_2_) and 5% CO_2_ for 24 h, and 48 h (for optimizing experiments also 72 h, 96 h and 120 h). Cells were collected for RNA isolation, proliferation (WST-1 assay), cell viability (AnnexinV/PI staining), cell cycle kinetics (BrdU staining) and metabolic assays.

### Chemically induced hypoxia

Chemically induced hypoxia was made in vitro through the addition of deferoxamine mesylate salt—DFO (#D9533, Sigma-Aldrich) and dimethyloxalylglycine, N-(Methoxyoxoacetyl)-glycine methyl ester – DMOG (#D3695, Sigma-Aldrich) to a well containing 2 × 10^6^ cells. Cells were cultured in IMDM medium (LM-I1090, Biosera) supplemented with 10% of fetal bovine serum (#FB-1090, Biosera) and 1% of P/S (#P4333, Sigma Aldrich). Cells were then incubated for 3 h, 6 h, and 24 h at 37 °C in 5% CO_2_ with DFO (final concentration 200 µM) and DMOG (final concentration 1 mM). After each time interval of incubation with DFO or DMOG, cells were collected, and total RNA was extracted for further gene expression detection. In parallel, cells were collected for flow cytometry measurement of apoptosis (AnnexinV/PI staining).

### Proliferation and cell viability tests

The WST-1 assay was used for the cell proliferation and viability measurement, followed by manufacturer’s protocol (ROCHE, #11 644 807 001). MEC-1 cells were seeded 5000 cells per well (used 96 well plate, flat bottom) in culture medium and incubated with 10 µL of WST-1 solution for 3 h [in normoxia and hypoxia conditions (1% O_2_)]. After 3 h, the substrate reaction absorbance was measured at 440 nm wavelength (blank 600 nm) by using Spark^®^ multimode microplate reader (TECAN) spectrophotometer (Tecan i-control, 1.10.4.0, infinite 200Pro). The absorbance of blank was subtracted from measured samples wavelength. Data are from three independent experiments. Data were analyzed using t-test, two-tailed, paired.

Cell growth curve was created by cell count determined by hemocytometer (counting-chamber). Cells were counted daily for one-week (in parallel normoxia vs hypoxia (1% O_2_) conditions). Seeding density of cells at day 0 was 10,000. Data are from three independent experiments (samples were done in quadruplicate). Data were analyzed using t-test, two-tailed, paired.

Annexin V/PI staining was performed for evaluation of apoptotic and dead cells. MEC-1 cells (1 × 10^6^ cells) were washed with 1 × PBS, resuspended in 1 × Annexin V binding buffer, stained by 5 µL of Annexin V (15 min at RT) (FITC, # BMS500FI-300, Invitrogen). Cells were washed with 1 × PBS and kept on ice until measurement on flow cytometer. Shortly before measurement 5 µL of PI (# BMS500FI-300, Invitrogen) was added. Data were evaluated by Diva software, the software FlowJo was used for data visualization, and the measurement was performed with the use of the FACS Canto II BD flow cytometer (30 000 events).

### Cell cycle measurement by BrdU flow kit

For BrdU staining, 2 × 10^6^ cells/well was used. A volume of 30 uL of BrdU were added to the MEC-1 cell culture (final concentration 1 µM) and stained for 20 min at 37 °C in 5% CO_2_ and 21% O_2_ under normoxic and under hypoxic (1% O_2_) conditions as followed by the manufacturer’s protocol (# 552598, BrdU flow kit, BD). Before flow cytometry measurement, cells were stained with 7AAD (provided by BrdU kit, 20 µL/tube) for 10 min on ice. Measurement was performed on cytometer FACS Canto BD (50 000 events). The software FlowJo was used for data visualization. Data are from three independent experiments. Data were analyzed using t-test, two-tailed, paired.

### RNA isolation and qRT-PCR

Total RNA including microRNA was extracted from MEC-1 cells (2 × 10^6^) by TRI reagent (#TR118, MRC) with slight modification as over night precipitation with isopropanol at − 20 °C and followed by manufacturer’s protocol. An amount of 100 ng of total RNA including microRNA was reverse-transcribed by High-Capacity cDNA Reverse Transcription Kit with RNase Inhibitor (#4,374,966, ThermoFisherScientific). TaqMan-based PCR with specific probes (Universal Probe Library, ROCHE and ThermoFisherScientific primers designed with probes) was performed on QS7 Pro instrument (ThermoFisherScientific). As reference genes, *GAPDH* (for mRNA) and *RNU44* (for miRNA) were used. The miRNA/mRNA expression was calculated by 2 delta-delta CT algorithm from target and reference CT values [specific (s) and control (c) amplicons calculated by 2^ − (CTc-CTs)^ equation] [26]. Data were acquired using t-test, two-tailed, paired (*p < 0.05; **p < 0.01; ***p < 0.001; ****p < 0.0001).

### Western blot

Cells were lysed (2 × 10^6^) in RIPA buffer and sonicated (1 cycle, 10” at 40% power). Four micrograms of proteins were separated by 1-D polyacrylamide gel electrophoresis (Mini-PROTEAN^®^ TGX™ Precast Gels, Bio-Rad). Proteins were transferred to Immuno-Blot^®^ PVDF Membrane (#1,620,174, Bio-Rad) using Trans-Blot^®^ Turbo^™^ Transfer System (Bio-Rad) and probed with primary antibody overnight at 4 °C (anti-HIF-1α, sc-10790; anti-GAPDH sc-51907, Santa Cruz Biotechnology). The following day, the membrane was washed with 1 × TBS and probed with secondary anti-rabbit antibody (#A0545, Sigma-Aldrich), conjugated with horseradish peroxidase and detected using detection kit (#1,705,060, Clarity^™^ Western ECL Substrate, Bio-Rad). Signalling was detected using ChemiDoc MP Imaging System (Bio-Rad).

### Metabolic assays

*Mitochondria isolation* was followed by the referred protocol in [27]. Mitochondria protein content was measured by the BCA kit (#BCA1-1KT, Sigma-Aldrich) on Biotek Synergy HT Microplate Reader at 562 nm wavelength. The concentration of protein was calculated using a standard curve prepared using the BSA protein standards.

*Electron transport chain complex I to complex III* was followed protocol in [27]. Mitochondria protein content was measured by the BCA kit (#BCA1-1KT, Sigma-Aldrich). Absorbance (reduction from succinate to cytochrome C) was read at 550 nm wavelength for 6 min (1 read/15 s) on Biotek Synergy HT Microplate Reader. Results were calculated according to Lambert–Beer equation.

*The Glucose Uptake-GloTM Assay* (#J1341, Promega) was used according to the manufacturer´s manual. The MEC-1 cells were cultured in normoxia and hypoxia (1% O_2_, 24 h, and 48 h), then counted and 10,000 cells were transferred to a non-translucent 96-well cell culture plate. The reaction solution from the kit was added and the luminescence signal was acquired at 0.5 s on the Spark^®^ multimode microplate reader (Tecan i-control, 1.10.4.0, infinite 200Pro) and is shown as counts/s.

*2-NBDG* (2-(N-(7-Nitrobenz-2-oxa-1,3-diazol-4-yl)Amino)-2-Deoxyglucose)(2-DG) (#N13195, ThermoFisherScientific) was used for the measurement of glucose uptake in MEC-1 cells (ctrl and miR-155 deficient) as followed by the manufacturer´s recommendations. The MEC-1 cells were counted and 200,000 cells were seeded on 6-well plate (in triplicates) and then cultured in normoxia and hypoxia (1% O_2_) for 24 h and 48 h. Cells were washed with 1 × PBS, resuspended in 1 mL of Seahorse XF DMEM medium (#103,575–100, Agilent), and then incubated 10 min with 2-DG in final concentration 10 µM (in normoxia and in hypoxia). Next, cells were washed with 1xPBS and the cell pellet was resuspended in 200 µL of 1xPBS, followed by flow cytometry. Shortly before measurement, 5 µL of PI (# BMS500FI-300, Invitrogen) were added. As 2-DG has an excitation/emission maximum of ∼465/540 nm, we used channel FITC 530 in this case. Data were evaluated by Diva software and the measurement was performed with the use of the FACS Canto II BD flow cytometer (50 000 events).

*The Lactate Assay* (#MAK064, Sigma-Aldrich) was used according to the manufacturer´s manual. The MEC-1 cells were cultured in normoxia and hypoxia (1% O_2_, 24 h, and 48 h), were then centrifuged (350´ g, RT, 5 min), and 10 µL of supernatant were aspirated for the assay and transferred to 96-well plate. Each sample was brought to a final volume of 50 µL/well with Lactate Assay Buffer. The reaction solution from the kit was mixed and 50 µL of Master Reaction Mix were added to each sample. Cells in the 96-well plates were incubated for 30 min at RT protected from light. Colorimetric absorbance was measured on Biotek Synergy HT Microplate Reader at 570 nm wavelength. Concentration of lactate was calculated using standard curve.

*mtATP* level was obtained by the Adenosine 5′-triphosphate (ATP) Bioluminescent Assay Kit (#FLAA, Sigma Aldrich). The MEC-1 control group and the MEC-1 miR-155 deficient cells (2 × 10^5^ cells) were cultivated 24 h and 48 h under the normoxia and hypoxia (1% O_2_) conditions. Cells were cultured under standard cell culture conditions. Mitochondria were isolated and further mtATP measurement was carried out according to the manufacturer´s protocol. Lumenescence was measured by the Biotek Synergy HT Microplate Reader.

*ATP* level was determined by the CellTiter-Glo^®^ 2.0 Cell Viability Assay (#G9242 Promega). The MEC-1 ctrl and the MEC-1 miR-155 deficient cells were cultivated 24 h and 48 h under the normoxia and hypoxia (1% O_2_) conditions. Cells were cultured under standard cell culture conditions and 5,000 cells were used for measurement. The assay was performed according to the manufacturer's manual. The luminescence signal was acquired at 0.5 s on the Spark^®^ multimode microplate reader (Tecan i-control, 1.10.4.0, infinite 200Pro) and is shown as counts/s.

*LDH level* was acquired by the LDH-Glo^™^ Cytotoxicity Assay (#J2380 Promega). The MEC-1 ctrl and the MEC-1 miR-155 deficient cells were cultivated 24 h and 48 h under the normoxia and hypoxia (1% O_2_) conditions. Cells were cultured under standard cell culture conditions and 2 µL of the cultivation media out of 1 ml was used for the measurement. The assay was performed according to the manufacturer´s manual. The luminescence signal was acquired at 0.6 s on the Spark^®^ multimode microplate reader (Tecan i-control, 1.10.4.0, infinite 200Pro).

*Pyruvate* concentration was determined by the Pyruvate Assay Kit (#MAK332 Sigma-Aldrich). The MEC-1 control group and the MEC-1 miR-155 deficient cells were cultivated 24 h and 48 h under the normoxia and hypoxia (1% O_2_) conditions. Cells were cultured under standard cell culture conditions and 1 × 10^6^ were homogenized mechanically in 1 ml of cold PBS; 10 µl were used for the measurement. The assay was performed according to the manufacturer´s manual. The fluorescence signal was acquired, excited by 530/20 nm and emission was read at 585/20 nm on the Spark^®^ multimode microplate reader (Tecan i-control, 1.10.4.0, infinite 200Pro).

## Results

### Optimization of the oxygen level and time points for in vitro culturing of MEC-1 cells in hypoxia

**Fig. 1:**
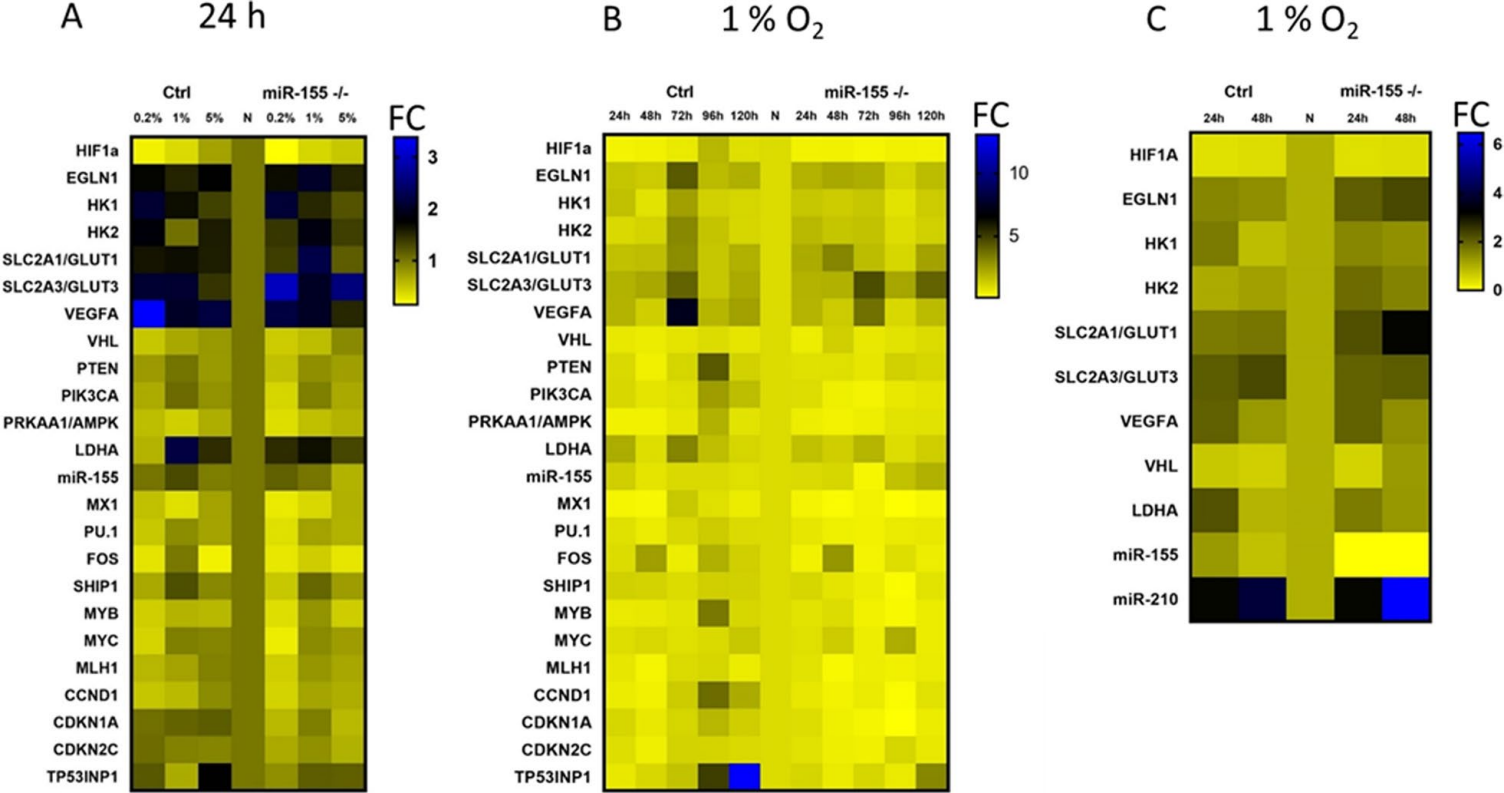
Optimization of the oxygen level in in vitro culturing of MEC-1 cells based on gene expression profiling. Heat maps show the gene expression (TaqMan-based qRT-PCR) profiles of MEC-1 cells (control (Ctrl) and miR-155 deficient clone #48 (miR-155^–/–^)) in normoxia vs hypoxia. **A** Heat map shows changes (as Fold Change, FC) in the mRNA/miRNA expression level in different hypoxia conditions. Hypoxia conditions were set as follows: 0.2, 1 and 5% O_2_ for a period of 24 h. **B** Heat map shows changes in the mRNA/miRNA expression level in 1% O_2_ (hypoxia) in MEC-1 ctrl and miR-155 ^–/–^ cells in different time-periods (24 h–120 h). **C** Heat map summarizes the expression level of selected hypoxia-related genes measured in the final hypoxia and time-period settings in MEC-1 cells, 1% and 24 h–48 h, respectively. In all 3 heat maps, the expression data were normalized to the expression level measured in normoxia and set as 1. The gene expression in all heat maps is shown as fold change (FC) and its intensity expresses the color scale next to each heat map

**Fig. 2:**
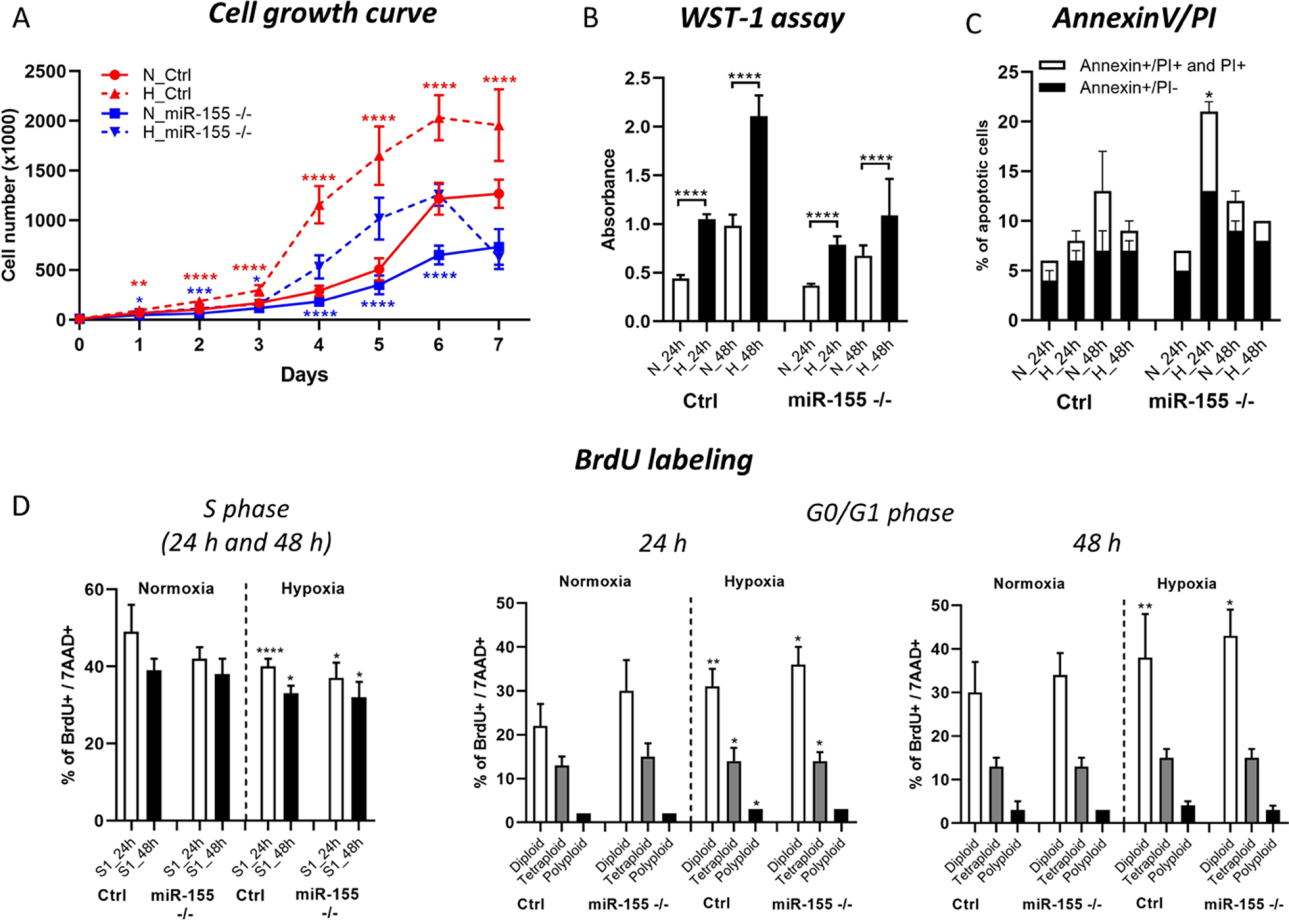
Proliferation and cell viability of MEC-1 cells during hypoxia. **A** The cell growth curve of MEC-1 cells (ctrl and miR-155^–/–^) in normoxia (N) vs hypoxia (H). Cells were counted within 7 days in hemocytometer chamber. For statistics we compared cell numbers in MEC-1 control cells in normoxia vs in hypoxia and the same for miR-155 deficient MEC-1 cell line. **B** Cell viability was measured by WST-1 assay in both normoxia vs hypoxia in 24 h and 48 h time periods. **C** Percentage of apoptotic cells measured by flow cytometry after Annexin V/PI staining. Cells were stained in parallel in normoxia and hypoxia conditions in 24 h and 48 h time periods. **D** Detection of the cell cycle kinetics by BrdU labelling in normoxia vs hypoxia in 24 h and 48 h. Left graph shows percentage of BrdU/7AAD positive cells. Right graphs depict cell population in G0/G1 phase. Representative picture of dot plot is in Supplemental Figure 3. For statistics we compared percentage (populations) of MEC-1 control cells in normoxic vs MEC-1 control cells in the hypoxic conditions (the same for miR-155 deficient MEC-1 cells). All values are means ± standard deviation obtained from three independent experiments. Statistics: t-test, two tailed, paired was used (*p<0.05; **p<0.01; ***p<0.001; ****p<0.0001). Additionally, we conducted the statistical analysis of MEC-1 ctrl cells vs miR-155^–/–^ in normoxia and so on in the hypoxia for the whole Figure 2. The statistical significance is shown in the Supplemental Material/Tables.

Due to a relatively wide interval of oxygen level, in the normal tissues between 1–11% and in the tumor about 2% [28] and lack of consistent hypoxia experimental data, we started with optimizing the oxygen level and time periods for further hypoxia experiments. First, we cultured MEC-1 cells (ctrl and miR-155^–/–^) in different oxygen levels (0.2%, 1% and 5%) in the hypoxic glove box. The final oxygen level was set up by following criteria: high expression of hypoxia related genes (*HIF1α, EGLN1, LDHA, VEGFA, GLUT1, GLUT3, HK1, HK2*) and low expression of *TP53INP1*. As the highest expression level of *LDHA, HK1, GLUT1, GLUT3* and the lowest expression of the *TP53INP1* (the anti-proliferative and pro-apoptotic protein) were detected in the 1% O_2,_ this O_2_ concentration was determined to be the most suitable for further experiments (Fig. 1A). Next, we optimized the time period for incubation of MEC-1 cells in 1% O_2_ hypoxia. With prolonged incubation of cells in hypoxia the level of apoptosis increased (Figs. 2C and 1B), so the best time period based on the expression level (low) of *TP53INP1* mRNA was set up for 24 h and 48 h (Fig. 1B). Figure 1C contains the final selection of the gene expression profile detected under hypoxia (1% O_2_) within 24 h and 48 h time periods. Our gene expression data showed extremely elevated levels of a well-known hypoxamiRNA, miR-210, in MEC-1 cells in hypoxia (1% O_2_) (Fig. 1C). The elevated level of miR-210 served as an additional internal control of presence of hypoxia in cells. The highest mRNA level showed hypoxia-related genes such as *EGLN1, GLUT1, GLUT3, VEGFA, LDHA,* and *HK1*. The lower level of miR-155 in MEC-1 control cells is probably the result of adaptation of the cells to the hypoxic condition (Fig. 1C). The general low expression level of *HIF1α* (also at the protein level, Supplemental Fig. 2) could be caused by its low stability and fast degradation within hours in hypoxia [39]. Another possible reason could be the high level of *EGLN1* mRNA (as its primary function is to degrade HIF1α protein) and low level of *VHL* or another secondary effect of miRNAs regulatory pathway on the HIF family [40].

### MiR-155 deficiency results in the lower cell proliferation

Hypoxia in general stimulates leukemic and cancer cells proliferation. Here we asked if the deletion of miR-155, oncomiRNA and probable hypoxamiRNA impairs the proliferation of MEC-1 cells in hypoxic conditions. Usually, hypoxia stimulates the proliferation of cells. Here we observed the opposite effect in relation to miR-155 expression. This observation was in concordance with our hypothesis, where we assumed superior effect of miR-155 expression toward hypoxia in the leukemic MEC-1 cells. Here we detected that the cell proliferation significantly decreased in miR-155 deficient MEC-1 cells under both oxygen conditions, in comparison to the MEC-1 control cells (Fig. 2A, B; and Supplemental Material/Tables). Decreased cell proliferation is mediated by miR-155 expression in miR-155 deficient MEC-1 cells, confirmed by proliferation curve and WST-1 assay (Fig. 2A, B). The significant difference in the cell proliferation was visible from day 4 (Fig. 2A). We also assessed the statistical difference between the MEC-1 control and MEC-1 miR-155 deficient cells in different oxygen conditions (normoxia and hypoxia separately) (Supplemental Material/Tables). Deficiency of miR-155 resulted in the increased number of apoptotic cells (Fig. 2C). Next, for assessment of the cell cycle kinetics, we labeled MEC-1 cells by BrdU. BrdU labeling points out significantly lower percentage of cells in the S phase, more distinct in miR-155 deficient MEC-1 cells (Fig. 2D, left graph). Hypoxia enriched the cell population in G0/G1 phase more prominently in miR-155^-/-^ MEC-1 cells (Fig. 2D, right graphs). The statistical significance is shown in Supplemental Material/Tables.

In conclusion, hypoxia significantly stimulates proliferation of MEC-1 cells depending on the miR-155 expression. MiR-155 is important for cell growth, and its absence even in hypoxia results in the lower proliferation of miR-155 deficient MEC-1 cells with increased level of apoptosis in comparison to control MEC-1 cells.

### Chemically-induced hypoxia stimulates hypoxia-related genes and hypoxamiR-210 in MEC-1 cells

**Fig. 3:**
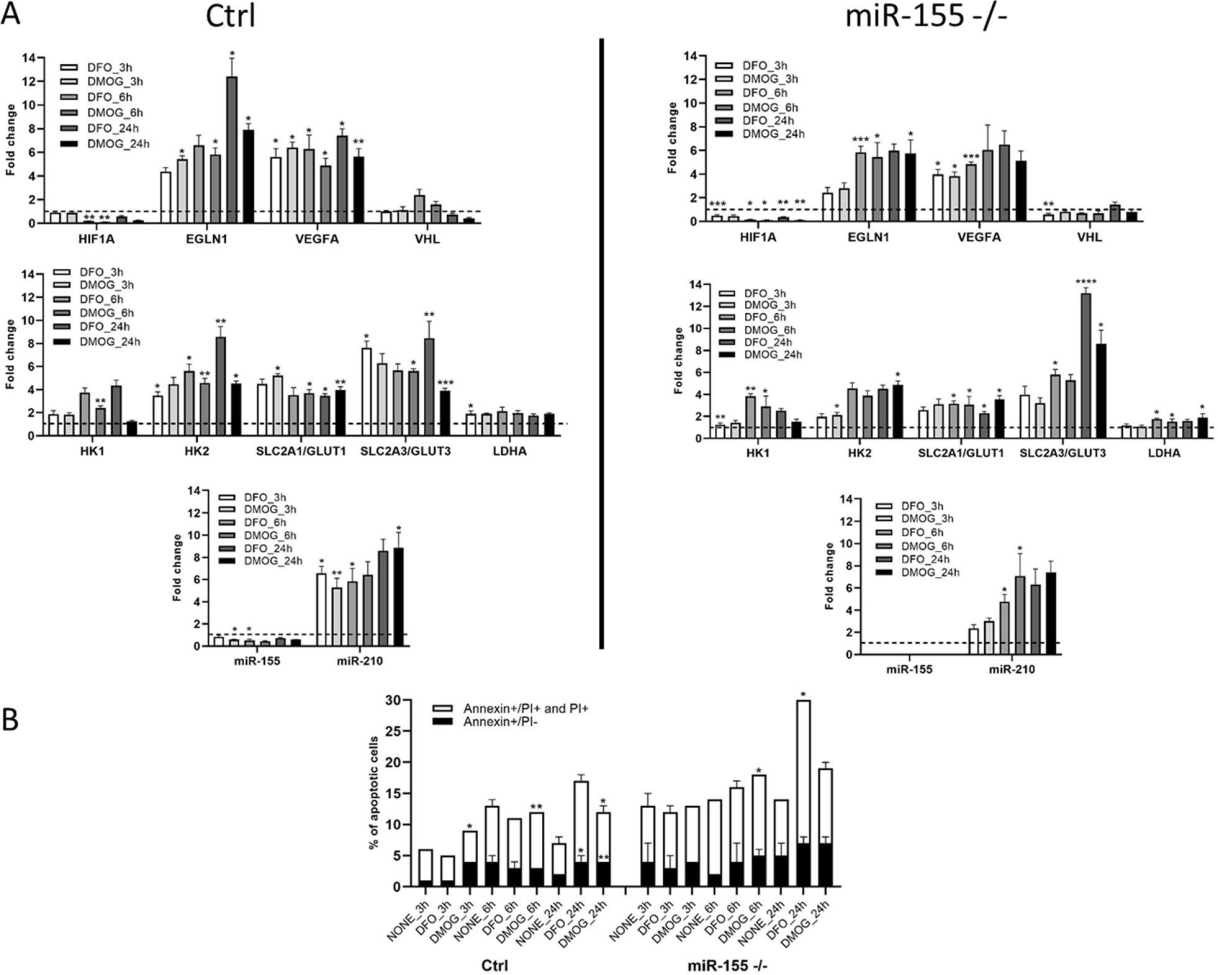
Chemically-induced hypoxia in vitro in MEC-1 cells.** A** MEC-1 cells (control (ctrl) left part of panel **A**; miR-155 deficient right part of the panel (**A**) were treated with chemicals DFO and DMOG for 3 h, 6 h and 24 h that induce total hypoxia in cells in vitro. Gene expression profile contains the same set up of genes as in hypoxia experiments performed in the hypoxia glove box. In all graphs, expression data were normalized to expression level in normoxia and set as 1. **B** Cell viability of MEC-1 cells after chemically-induced hypoxia was assessed by flow cytometry measurement of Annexin V/PI. Cells were stained in parallel in normoxia (NONE) and in chemically-induced hypoxia in 3 h, 6 h and 24 h time periods. All values are means ± standard deviation obtained from three independent experiments. We compared values obtained in untreated MEC-1 cells (normoxia, NONE) vs chemically treated by DFO or DMOG for each time period and cell line (ctrl, miR-155^–/–^ separately). Statistics: t-test, two tailed, paired was used, (*p < 0.05; **p < 0.01; ***p < 0.001; ****p < 0.0001)

We chemically-induced hypoxia with DFO and DMOG in MEC-1 cells to validate the data from the hypoxia glove box. Chemical inducers of hypoxia DFO and DMOG operate rapidly, thus we selected short time periods as 3 h, 6 h and 24 h for their assessment (Fig. 3A). We measured the same set of genes as in Fig. 1C. Expression levels of *EGLN1*, *GLUT3* and *VEGFA* mRNA showed the highest fold change in both control and miR-155 deficient MEC-1 cells (Fig. 3A). The gene *GLUT3* showed significantly highest expression level after 24 h in miR-155 deficient MEC-1 cells (after DFO + 13,0 FC; after DMOG + 8,0 FC) in comparison to MEC-1 control cells (after DFO + 8,0 FC; after DMOG + 4,0 FC) (Fig. 3A). Interestingly, in the hypoxia glove box the *GLUT3* reached much lower expression (+ 3,0 FC in control and + 2,0 FC in miR-155^–/–^, 48 h, Fig. 1C) and surprisingly the *GLUT1* showed the highest expression level in miR-155 deficient MEC-1 cells (+ 5,0 FC, in 48 h, Fig. 1C). As expected, chemically-induced hypoxia significantly increased the expression of well-known hypoxiamiRNA-210 in both control (after 24 h of DFO + 8,0 FC and of DMOG + 8,2 FC) and miR-155 deficient MEC-1 cells (after 24 h of DFO + 6,2 FC and of DMOG + 7,8 FC; Fig. 3A). Similarly, we detected upregulation of miR-210 in hypoxia performed in the hypoxia glove box; for MEC-1 control (24 h + 4,0 FC; 48 h + 5,0 FC) and for miR-155^–/–^ MEC-1 cells (24 h + 4,0 FC; 48 h + 6,0 FC) (Fig. 1C). The expression of miR-155 in MEC-1 control cells in both, chemically-induced hypoxia and hypoxia in the hypoxia glove box did not increase much (Fig. 1C and Fig. 3A). Both chemical inducers of hypoxia showed similar trends in the gene expression level of selected genes. As these chemicals induced the total hypoxia in cells, we measured the level of apoptosis during all selected time periods in MEC-1 cells. Based on the flow cytometry data from measurement of Annexin V/PI we could conclude that in the abovementioned conditions the chemically-induced hypoxia caused overall slightly increased level of apoptosis in MEC-1 control cells. Overall higher apoptosis was detected in the miR-155 deficient MEC-1 cells, with a peak after 24 h time period (~ 28% after DFO) (Fig. 3B). In summary, the chemically-induced hypoxia vs hypoxia performed in the hypoxia glove box in MEC-1 cells showed similar trend in some genes. The most significant change was detected in the expression of *GLUT3. GLUT3* mRNA showed + 13,0 FC increasement after DFO and + 8,0 FC after DMOG (24 h time period) in miR-155 deficient MEC-1 cells. Also, miR-155 deficient MEC-1 cells seem to be more sensitive to apoptosis in comparison to MEC-1 control cells.

### Cellular metabolism of leukemic cells during hypoxia significantly depends on the presence of miR-155

**Fig. 4:**
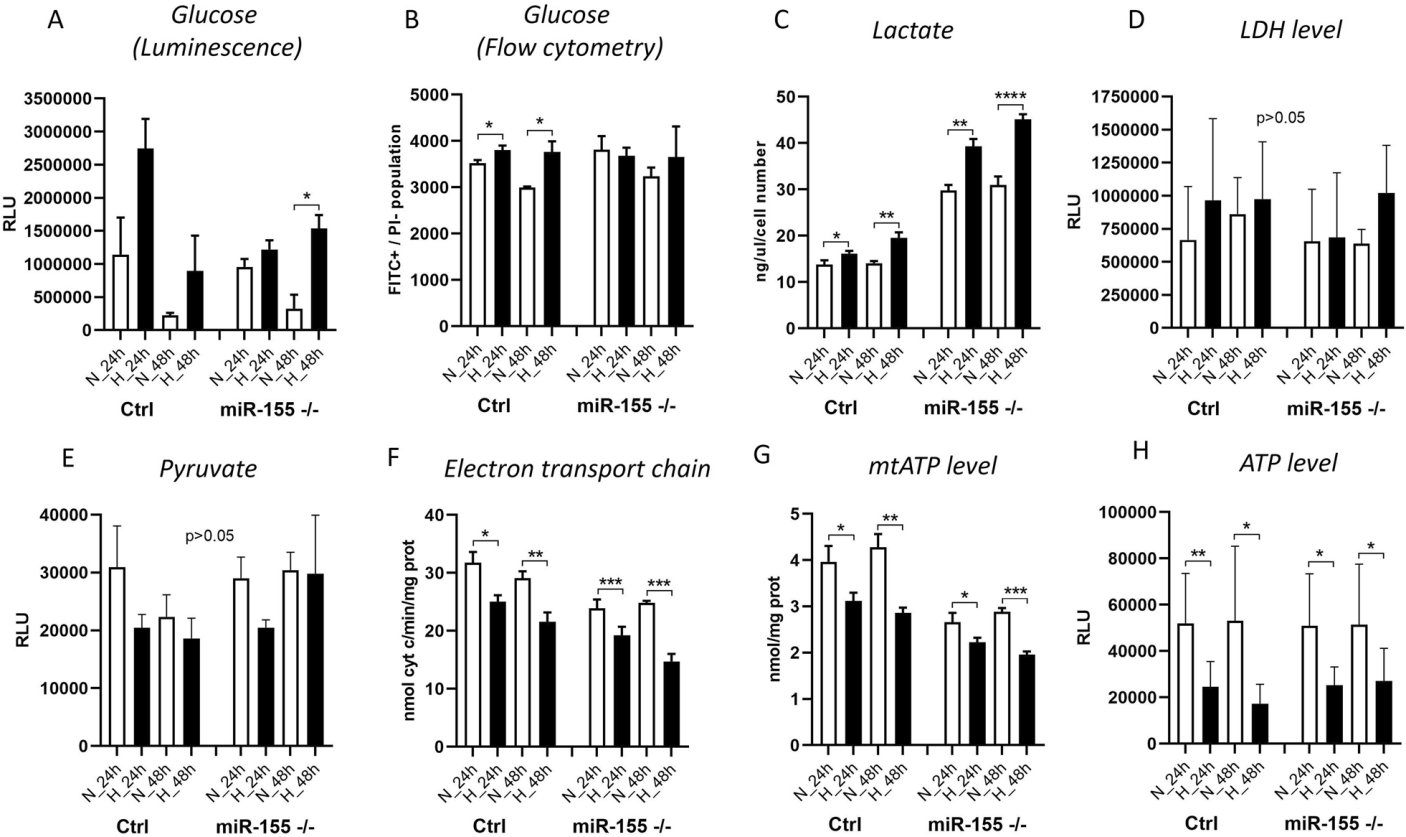
Measurement of metabolism in MEC-1 cells in normoxia and hypoxia. For the assessment of glucose uptake in MEC-1 cells (control (ctrl) and miR-155 ^–/–^) in normoxia vs hypoxia we used **A** Glucose Uptake-GloTM Assay and **B** 2-NBDG fluorescent molecule. **C** Shows the concentration of Lactate measured by colorimetric assay. **D** LDH level was measurement by LDH-Glo^™^ Cytotoxicity Assay. **E** Pyruvate concentration was measured by Pyruvate Assay Kit. **F** The electron transport chain form complex I to complex III (ETC) was measured by following the protocol in [20]. **G** mtATP level was measured by Adenosine 5′-triphosphate (ATP) Bioluminescent Assay Kit. **H** ATP level was measured by CellTiter-Glo^®^ 2.0 Cell Viability Assay. All values are means ± standard deviation obtained from three independent experiments. Statistics t-test, two-tailed, paired was used, (*p < 0.05; **p < 0.01; ***p < 0.001; ****p < 0.0001)

In oxygen-deprived conditions leukemic cells shift their cellular metabolism from oxidative phosphorylation to glycolysis. This results in increased glucose uptake and significant overall changes in metabolism. Here we asked whether absence of miR-155 in MEC-1 cells during hypoxia also leads to higher glucose uptake. Interestingly, miR-155 deficiency led to increase of glucose uptake in hypoxia, significant only in miR-155 deficient MEC-1 cells after 48 h (Fig. 4A). Additionally, we performed measurement of 2-deoxy-glucose (2-NBDG) by flow cytometry, and we observed a similar trend of increased glucose uptake. Data show an increased glucose uptake by MEC-1 cells in hypoxia, significantly only in MEC-1 control cells, while in the miR-155 deficient cells we did not observed much change (Fig. 4B). From our preliminary data from mRNA-seq (not shown), we know that miR-155 deficiency results in overexpression of *LDHA* gene that encodes the last enzyme of anaerobic glycolysis. Here we detected overexpression of *LDHA* mRNA by qRT-PCR (Fig. 1A, C). As expected, we found the level of lactate increased in hypoxia; this is in line with the effect of miR-155 deficiency on LDHA levels where the production of lactate increased more rapidly in miR-155 deficient MEC-1 cells (Fig. 4C), suggesting an enhanced lactate metabolism. We also measured the level of lactate dehydrogenase (LDH) by luminescence assay, which appeared to have a tendency to rise in hypoxia (Fig. 4D). On the contrary, the level of pyruvate during hypoxia decreased (Fig. 4E). While the mitochondria represents a key organelle of energetic metabolism, their efficiency is turned off in hypoxia [38]. Here we additionally measured the activity of Electron Transport Chain (ETC) and mitochondrial (mt)ATP and ATP in MEC-1 cells under hypoxia. We observed that hypoxic MEC-1 cells had reduced ETC (Fig. 4F), mtATP (Fig. 4G) and ATP (Fig. 4H). Notably, the decrease in these parameters was strikingly higher in the miR-155-deficient cells (Fig. 4F–H). This all points to the rapid changes in the cellular metabolism of MEC-1 cells under hypoxia, significantly depending on the presence of miR-155, whose deficiency accelerates the lactate metabolism elicited by hypoxia.

## Discussion

Hypoxia is a hallmark of cancer and is a hematopoietic niche that maintains proliferation of cells. Hypoxia modulates cell cycle, transcriptome and cellular metabolism. The metabolic response to hypoxia results in a shift of ATP production to glycolysis and lactate metabolism at the expense of oxidative phosphorylation [28]. In this study, we aimed to describe how oncogenic miR-155 influences CLL cells in hypoxia in terms of gene expression profiling (mRNA/miRNA expression level). In general, there is not a uniform and exact opinion among researchers on the oxygen level (percentage) in the leukemic niche and its application for in vitro conditions in laboratory. From literature, it is known that the oxygen level in normal tissues varies between 1 and 11% and in tumors is about 2% [29]. The B cells are characterized by intensive migration and circulation in the human body, from blood stream with almost normoxia into tissues (spleen, lymph nodes) with different hypoxia levels [30]. In line with this, Koczula et al., 2016 performed a detailed analysis of oxygen levels in CLL cells in vivo by nuclear magnetic resonance (NMR) technique [4]. They described that CLL cells are very flexible in different oxygen levels and could rapidly adapt by modulating their transcriptome and metabolome [4]. The main message of this work with primary CLL cells points to very heterogeneous oxygen levels in the human body and extreme plasticity of CLL cells. CLL cells migrate (blood stream, lymph nodes, spleen, bone marrow) from almost normoxia in the circulating blood into lymph nodes where oxygen is about 3%. In order to determine the best range of oxygen levels that will be used in further experiments, we optimized the oxygen level (0.2%, 1% and 5%) and time periods (24 h–120 h) (Fig. 1A, B). Based on the highest expression level of hypoxia-related genes as *LDHA, HK1, GLUT1, GLUT3* and the lowest expression of pro-apoptotic gene *TP53INP1* we found the optimal in vitro hypoxic condition is at 1% oxygen and in the period of 24 h and 48 h (Fig. 1C). Similarly, Koczula et al., 2016 confirmed hypoxia by overexpression of mRNAs of *LDHA*, *VEGF* and *GLUT1* in primary CLL cells [4]. In the blood stream, the CLL cells are used to hypoxia and are characterized by high *HIF-1α* expression when entering into lymphoid tissues [4]. In lymphoid tissues, the CLL cells are constantly supplied by essential signals for their survival and proliferation through interaction with accessory cells, usually stromal cells, contributing to drug resistance and apoptosis [31]. The *HIF-1α* expression is induced also by PI3K and ERK mitogen-activated protein kinase (MAPK) signaling in stromal cells [32]. Surprisingly, we were not able to detect increased level of *HIF1α* at mRNA (Fig. 1C) and at the protein level (Supplemental Fig. 2). We assume that it could be due to the increased mRNA level of miR-155 (in MEC-1 control cells in hypoxia), *EGLN1, PIK3CA* and low level of *VHL* as these are the key regulators of *HIF1α* and direct targets of miR-155 [23] or by another miRNA impairment. Another possible reason could be fast degradation of HIF1α during the first 24 h of hypoxia [39]. In addition to transcription factors and other molecules, hypoxia modulates microRNAs, which react by their extreme expression. Among the well-described hypoxamiRNAs belongs miR-210 [17]. MEC-1 cells react to hypoxia with a significantly elevated level of miR-210 (Figs. 1C and 3A) that is in concordance with other observations [17] and reviews [33]. Another explanation for why we were not able to detect HIF1α could be its replacement by HIF-2 signaling after longer exposure of cells to hypoxia [17]. Hypoxic conditions could be induced experimentally by using different methods and molecules [34]. To validate the in vitro hypoxia accomplished in the hypoxic glove box we induced hypoxia in CLL (MEC-1) cells chemically. Thus, MEC-1 cells were treated with 2‐OG analogue, dimethyloxalylglycine (DMOG), a competitive inhibitor of prolyl hydroxylase domain‐containing proteins and with deferoxamine mesylate salt (DFO), an iron-chelating agent, both for 3 h, 6 h, and 24 h (Fig. 3A). Both chemicals significantly induced hypoxia in vitro in similar actions. Surprisingly, chemically-induced hypoxia resulted in more pronounced expression of hypoxia-related genes as *EGLN1, VEGFA, HK2* and *GLUT3* (Fig. 3A) with moderate level of apoptosis (Fig. 3B) in comparison to the data from hypoxic glove box (Fig. 1C). Increased level of apoptosis by chemical inductors of hypoxia is in accordance with others [35]. Increased cell proliferation in hypoxia, especially in cancer cells, is a common hallmark. Under normal conditions, cell proliferation decreases with low oxygen levels or stress. In addition, overexpression of *HIF-1α* results in cell cycle arrest in normal cells (lymphocytes, keratinocytes, embryonic stem cells, and hematopoietic stem cells) [36]. However, the opposite situation happens in the malignant cells. In general, malignant cells proliferate at a high rate and express high levels of *HIF-1α* not only due to hypoxia but also due to deregulated signaling pathways that increase their survival [16]. Here we detected significantly higher proliferation rates of MEC-1 cells in hypoxia depending on the presence of miR-155 (Fig. 2A, B) and accompanied by slightly increased apoptosis in the absence of miR-155 (Fig. 2C). One of the potential explanations could be deregulation of HIF-dependent miRNA regulatory network. Overexpression of *HIF-1α* induces the expression of oncomiRNAs in malignant cells [16]. Sawai et al., 2022 selected 17 hypoxia related miRNAs (hypoxamiRNAs) in solid tumors, among them also miR-155 and miR-210. Similarly, these miRNAs were significantly changed in MEC-1 cells (Fig. 1C and Fig. 3A), especially miR-210, which was highly upregulated. MiR-155 represents the key oncomiRNA and has several combined roles in preventing apoptosis, modulating gene expression, blocking the phosphorylation of glucose, promoting many cancers and leukemias [19]. There is evidence that miR-155 is actively involved in glucose metabolism in breast cancer, such that deletion of miR-155 abolishes glucose uptake [13]. However, according to our data, MEC-1 cells reacted to hypoxia by increasing glucose uptake (Fig. 4A, B). This discordance could be due to different cell origin, breast cancer versus CLL where miR-155 could work in a different manner. Furthermore, the direct target of miR-155 is *HK2* (hexokinase 2; by current MiRTarBase version 9.0, 2022; [37]) which phosphorylates glucose, thereby committing it to the glycolytic pathway. This is additional evidence that glucose metabolism is controlled by miR-155. We detected significantly increased levels of *HK2* mRNA in miR-155 deficient MEC-1 cells (Fig. 1A, C). The absence of miR-155 results in increased glucose uptake and lactate production in MEC-1 cells in hypoxia (Fig. 4A, B, C). This notion is in accordance with experiments performed on the primary CLL cells, where the production of lactate was correlated with the glucose consumption [4]. At the same time the mitochondrial oxidative phosphorylation was turned off in hypoxia and this effect was exacerbated by the deficiency of miR-155.

To conclude, CLL cells are highly flexible and can adapt to different oxygen levels by coordinated changes at the mRNA/miRNA expression level and metabolism. One possible mechanism of this adaptation could be through the upregulation of glucose transporters (e.g., *GLUT1* and *GLUT3*) that promotes glucose uptake and anaerobic metabolism and simultaneously shuts-down the mitochondrial energetic metabolism. These abovementioned effects are strengthened in the absence of miR-155. 

## Conclusions

In the present study, we aimed to answer questions regarding the role of miR-155 (in terms of proliferation, metabolism and gene expression profile) in MEC-1 cells under hypoxic conditions. We confirmed the coupled effect of hypoxia and absence of miR-155 on the proliferation rate of MEC-1 cells, where the deletion of miR-155 significantly inhibits cell growth in normoxia and in hypoxia. Hypoxia-related genes such as *EGLN1, VEGFA, HK2, HK1, LDHA* and especially *GLUT1, GLUT3* were upregulated in hypoxia. We detected the extreme upregulation of hypoxamiR-210 in MEC-1 cells under hypoxia. We showed the effect of miR-155 presence/absence on glucose and lactate metabolism by qRT-PCR and by metabolic assays. Interestingly, the absence of miR-155 in MEC-1 cells stimulate the expression of mRNA of *EGLN1, HK2, GLUT1* in the hypoxic glove box and *GLUT3* in the chemically-induced hypoxia, which could be one of the possible adaptation mechanisms at different levels of hypoxia.

### Supplementary Information

Supplementary Material 1. Figure S1: Description of creation of miR-155 deficient MEC-1 cells and its validation.

Supplementary Material 2. Figure S2: Immunoblot.

Supplementary Material 3. Figure S3: Representative dot plot pictures to all flow cytometry data within manuscript.

Supplementary Material 4. Tables.

## Data Availability

All data generated or analyzed during this study are included in this published article [and its supplementary information files].

## References

[1] Infantino V, Santarsiero A, Convertini P, Todisco S, Iacobazzi V (2021). Cancer cell metabolism in hypoxia: role of HIF-1 as key regulator and therapeutic target. Int J Mol Sci.

[2] Gacche RN, Assaraf YG (2018). Redundant angiogenic signaling and tumor drug resistance. Drug Resist Updat.

[3] Godet I, Shin YJ, Ju JA, Ye IC, Wang G, Gilkes DM (2019). Fate-mapping post-hypoxic tumor cells reveals a ROS-resistant phenotype that promotes metastasis. Nat Commun.

[4] Koczula KM, Ludwig C, Hayden R, Cronin L, Pratt G, Parry H, Tennant D, Drayson M, Bunce CM, Khanim FL (2016). Metabolic plasticity in CLL: adaptation to the hypoxic niche. Leukemia.

[5] Xie H, Simon MC (2017). Oxygen availability and metabolic reprogramming in cancer. J Biol Chem.

[6] Pliszka M, Szablewski L (2021). Glucose transporters as a target for anticancer therapy. Cancers.

[7] Wright EM (2013). Glucose transport families SLC5 and SLC50. Mol Asp Med.

[8] Long W, Panwar P, Witkowska K, Wong K, O'Neill D, Chen XZ, Lemieux MJ, Cheeseman CI (2015). Critical roles of two hydrophobic residues within human glucose transporter 9 (hSLC2A9) in substrate selectivity and urate transport. J Biol Chem.

[9] Riedl CC, Akhurst T, Larson S, Stanziale SF, Tuorto S, Bhargava A, Hricak H, Klimstra D, Fong Y (2007). 18F-FDG PET scanning correlates with tissue markers of poor prognosis and predicts mortality for patients after liver resection for colorectal metastases. J Nucl Med.

[10] Barron CC, Bilan PJ, Tsakiridis T, Tsiani E (2016). Facilitative glucose transporters: Implications for cancer detection, prognosis and treatment. Metabolism.

[11] Heydarzadeh S, Moshtaghie AA, Daneshpoor M, Hedayati M (2020). Regulators of glucose uptake in thyroid cancer cell lines. Cell Commun Signal.

[12] Taefehshokr S, Taefehshokr N, Hemmat N, Hajazimian S, Isazadeh A, Dadebighlu P, Baradaran B (2021). The pivotal role of MicroRNAs in glucose metabolism in cancer. Pathol Res Pract.

[13] Kim S, Lee E, Jung J, Lee JW, Kim HJ, Kim J, Yoo HJ, Lee HJ, Chae SY, Jeon SM (2018). microRNA-155 positively regulates glucose metabolism via PIK3R1-FOXO3a-cMYC axis in breast cancer. Oncogene.

[14] Li X, Yang Y, Zhang B, Lin X, Fu X, An Y, Zou Y, Wang J-X, Wang Z, Yu T (2022). Lactate metabolism in human health and disease. Signal Transduct Target Ther.

[15] Pedroza-Torres A, Romero-Córdoba SL, Justo-Garrido M, Salido-Guadarrama I, Rodríguez-Bautista R, Montaño S, Muñiz-Mendoza R, Arriaga-Canon C, Fragoso-Ontiveros V, Álvarez-Gómez RM (2019). MicroRNAs in tumor cell metabolism: roles and therapeutic opportunities. Front Oncol.

[16] Sawai S, Wong P-F, Ramasamy TS (2022). Hypoxia-regulated microRNAs: the molecular drivers of tumor progression. Crit Rev Biochem Mol Biol.

[17] Moszyńska A, Jaśkiewicz M, Serocki M, Cabaj A, Crossman DK, Bartoszewska S, Gebert M, Dąbrowski M, Collawn JF, Bartoszewski R (2022). The hypoxia-induced changes in miRNA-mRNA in RNA-induced silencing complexes and HIF-2 induced miRNAs in human endothelial cells. FASEB J.

[18] Faraoni I, Antonetti FR, Cardone J, Bonmassar E (2009). miR-155 gene: a typical multifunctional microRNA. Biochim Biophys Acta.

[19] Elton TS, Selemon H, Elton SM, Parinandi NL (2013). Regulation of the MIR155 host gene in physiological and pathological processes. Gene.

[20] Ferrajoli A, Shanafelt TD, Ivan C, Shimizu M, Rabe KG, Nouraee N, Ikuo M, Ghosh AK, Lerner S, Rassenti LZ (2013). Prognostic value of miR-155 in individuals with monoclonal B-cell lymphocytosis and patients with B chronic lymphocytic leukemia. Blood.

[21] Vargova K, Pesta M, Obrtlikova P, Dusilkova N, Minarik L, Vargova J, Berkova A, Zemanova Z, Michalova K, Spacek M (2017). MiR-155/miR-150 network regulates progression through the disease phases of chronic lymphocytic leukemia. Blood Cancer J.

[22] Tang L, Peng Y-Z, Li C-G, Jiang H-W, Mei H, Hu Y (2019). Prognostic and clinicopathological significance of MiR-155 in hematologic malignancies: a systematic review and meta-analysis. J Cancer.

[23] Ghosh AK, Shanafelt TD, Cimmino A, Taccioli C, Volinia S, Liu C-G, Calin GA, Croce CM, Chan DA, Giaccia AJ (2009). Aberrant regulation of pVHL levels by microRNA promotes the HIF/VEGF axis in CLL B cells. Blood.

[24] Griggio V, Vitale C, Todaro M, Riganti C, Kopecka J, Salvetti C, Bomben R, Bo MD, Magliulo D, Rossi D (2020). HIF-1α is over-expressed in leukemic cells from TP53-disrupted patients and is a promising therapeutic target in chronic lymphocytic leukemia. Haematologica.

[25] Kontos CK, Papageorgiou SG, Diamantopoulos MA, Scorilas A, Bazani E, Vasilatou D, Gkontopoulos K, Glezou E, Stavroulaki G, Dimitriadis G (2017). mRNA overexpression of the hypoxia inducible factor 1 alpha subunit gene (HIF1A): an independent predictor of poor overall survival in chronic lymphocytic leukemia. Leuk Res.

[26] Livak KJ, Schmittgen TD (2001). Analysis of relative gene expression data using real-time quantitative PCR and the 2(-Delta Delta C(T)) method. Methods.

[27] Wibom R, Hagenfeldt L, von Döbeln U (2002). Measurement of ATP production and respiratory chain enzyme activities in mitochondria isolated from small muscle biopsy samples. Anal Biochem.

[28] Sebestyén A, Kopper L, Dankó T, Tímár J (2021). Hypoxia signaling in cancer: from basics to clinical practice. Pathol Oncol Res.

[29] Lee P, Chandel NS, Simon MC (2020). Cellular adaptation to hypoxia through hypoxia inducible factors and beyond. Nat Rev Mol Cell Biol.

[30] Burrows N, Maxwell PH (2017). Hypoxia and B cells. Exp Cell Res.

[31] Hanna BS, Öztürk S, Seiffert M (2019). Beyond bystanders: myeloid cells in chronic lymphocytic leukemia. Mol Immunol.

[32] Seiffert M (2020). HIF-1α: a potential treatment target in chronic lymphocytic leukemia. Haematologica.

[33] Adhi Pangarsa E, Rizky D, Setiawan B, Santosa D, Mubarika Haryana S, Suharti C (2022). Crosstalk between hypoxia and inflammation in non-Hodgkin lymphoma. Bali Med J.

[34] Rinderknecht H, Ehnert S, Braun B, Histing T, Nussler AK, Linnemann C (2021). The art of inducing hypoxia. Oxygen.

[35] Guo M, Song L-P, Jiang Y, Liu W, Yu Y, Chen GQ (2006). Hypoxia-mimetic agents desferrioxamine and cobalt chloride induce leukemic cell apoptosis through different hypoxia-inducible factor-1alpha independent mechanisms. Apoptosis.

[36] Hubbi ME, Gilkes DM, Hu H, Kshitiz; Ahmed I., Semenza G.L.  (2014). Cyclin-dependent kinases regulate lysosomal degradation of hypoxia-inducible factor 1α to promote cell-cycle progression. Proc Natl Acad Sci U S A.

[37] Huang H-Y, Lin Y-C-D, Cui S, Huang Y, Tang Y, Xu J, Bao J, Li Y, Wen J, Zuo H (2022). miRTarBase update 2022: an informative resource for experimentally validated miRNA-target interactions. Nucl Acid Res.

[38] Panuzzo C, Pironi L, Maglione A, Rocco S, Stanga S, Riganti C, Kopecka J, Shahzad Ali M, Pergolizzi B, Bracco E, Cilloni D (2023). mTORC2 is activated under hypoxia and could support chronic myeloid leukemia stem cells. Int J Mol Sci.

[39] Koh M-Y, Powis G (2012). Passing the baton: the HIF switch. Trend Biochem Sci.

[40] Mircea I, Kaelin WG, Jr.  (2017). The EGLN1-HIF O_2_-sensing system: multiple inputs and feedbacks. Mol Cell.

