# Supplementary material for: MiR-155 deficiency and hypoxia results in metabolism switch in the leukemic B-cells
Source: Cancer Cell Int. 2024 Jul 18;24:251. doi: 10.1186/s12935-024-03437-8 (PMC11256420; doi:10.1186/s12935-024-03437-8)

**Supplemental Figure 1**

**CRISPR/Cas9 vector design and transfection of MEC-1 cell line**

Deletion of 23 nt sequence within the *MIR155HG* gene was performed using pU6gRNA-Cas9-GFP plasmid (#10021426MN, Sigma). Vector backbone was ordered from Sigma and targeted miR-155 sequence was designed *in-home*. An amount of 1 µg of vector DNA was used for the transfection of MEC-1 cell line using Amaxa Nucleofector II with B cell nucleofection kit (#VPA1001, Lonza) and a program for B cells, U-015. 24 hours post transfection the viable (Propidium Iodide, PI negative) and GFP positive cells (CRISPR plasmid contains sequence for GFP) were sorted out by FACS Aria II sorter BD (equipped with 489 nm (50 mW), 561 nm (100 mW), 638 nm (140 mW), 404 nm (100 Mw) and 355 nm (20 mW) lasers). Single cell clones were cultured in 96 well plates for 3 weeks followed by cryopreservation and DNA/RNA isolation for further test and experiments. The first test for the verification of the deletion of the desired sequence was done by Sanger sequencing of the PCR product (checked by PAGE elfo) that was performed on the single-cell clones that were expanded *in vitro* after sterile cell sorting. The second test was done by measurement of expression level of miR-155 by qRT-PCR. From the overall obtained 50 clones we found that only one, the clone 48, bears desired biallelic deletion of mature miR-155. Therefore the clone #48 was selected for the further experiments.


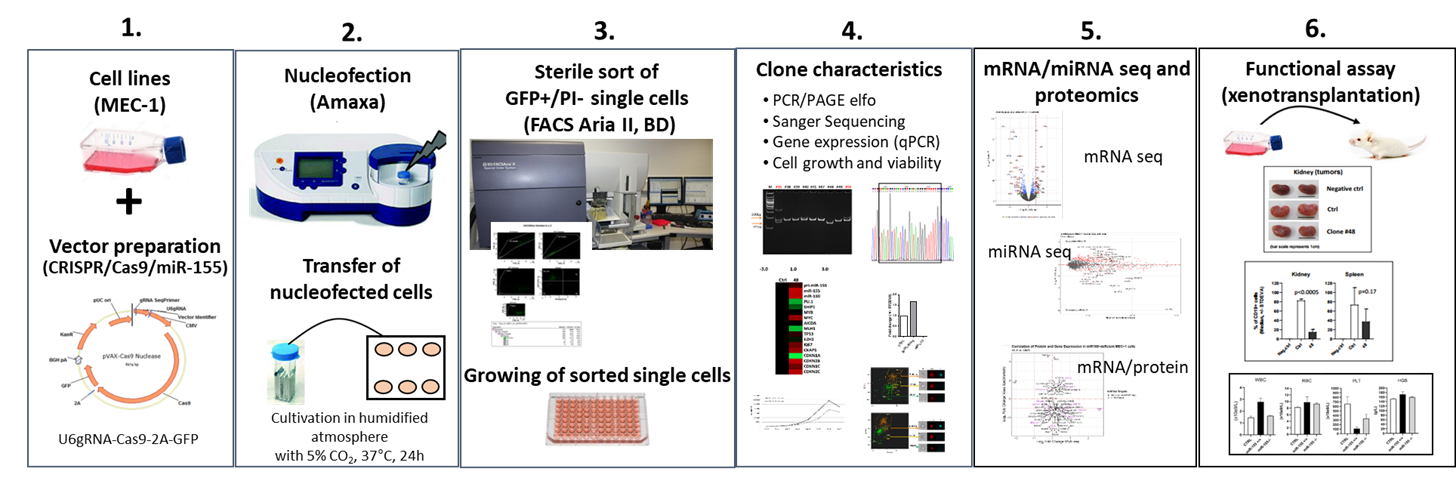


**Workflow of the generation of miR-155 deficient MEC-1 cell line/clone #48.** Panel 1 represents the first step, the MEC-1 cell line and CRISPR/Cas9 vector preparation. Panel 2 describes the introduction of CRISPR/Cas9/miR-155 vector by nucleofection into MEC-1 cells. Panel 3 shows sorting of the single cell clones based on the GFP expression and cell viability (PI staining). Panel 4 shows verification tests of miR-155 deletion (PCR/PAGE elfo, Sanger seq, qRT-PCR).

**
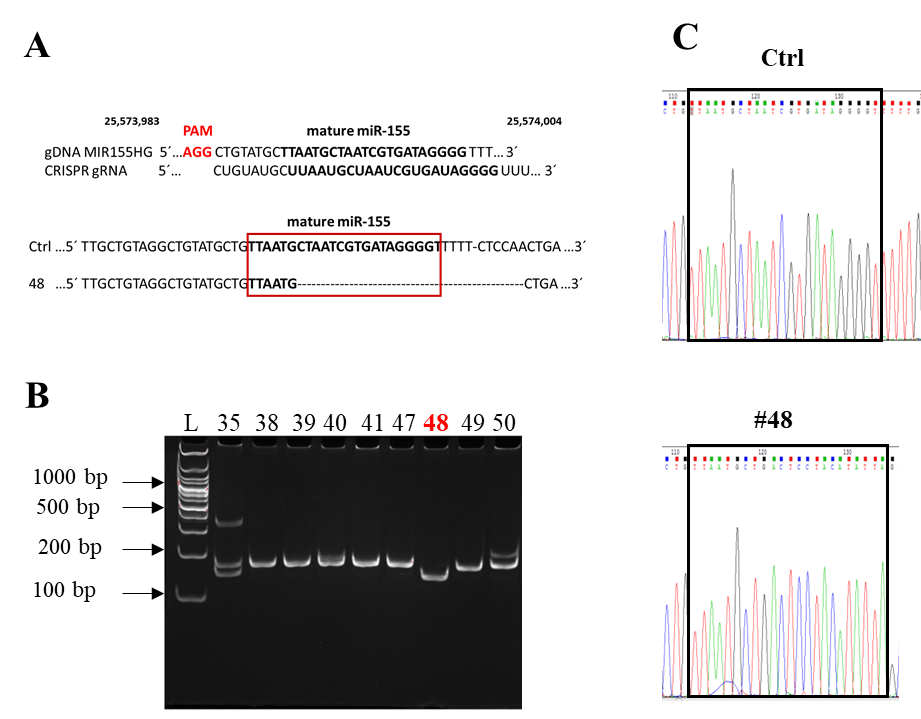
**

**Scheme of miR-155 editing by CRISPR/Cas9 in MEC-1 cells.** **A** The upper part of the picture shows the sequence of *MIR155HG* gene coding for mature miR-155 (in bold, 23 nt) that is the target sequence for gRNA with PAM sequence AGG shown in the red color. The lower part of the figure shows scheme with the region of *MIR155HG* gene from 5` 25,573,983 to 25,574,004 3` (NC_000021.9 (25,573,980 - 25,574,044), Chr 21, GRCh38.p13), where 16 nt are deleted in case of MEC-1 miR-155 deficient clone #48 (homozygous deletion) in comparison to ctrl (non-edited MEC-1 cells). **B** The PCR product of CRISPR clones (170 bp) detected on 10% PAGE gel. Clone #48 with biallelic deletion and proper size is highlighted in red color. **C** Chromatograms (Sanger sequencing, Chromas software for visualization of pictures was used) of the PCR product in MEC-1 cells (ctrl vs #48). The sequence responsible for mature miR-155 is highlighted in the black frame.

**Map of expression vector used for CRISPR/Cas9 gene editing - U6gRNA-Cas9-2A-GFP miR-155 (#10021426MN, Sigma).**

**Original datasheet/certificate of CRISPR vector/plasmid**


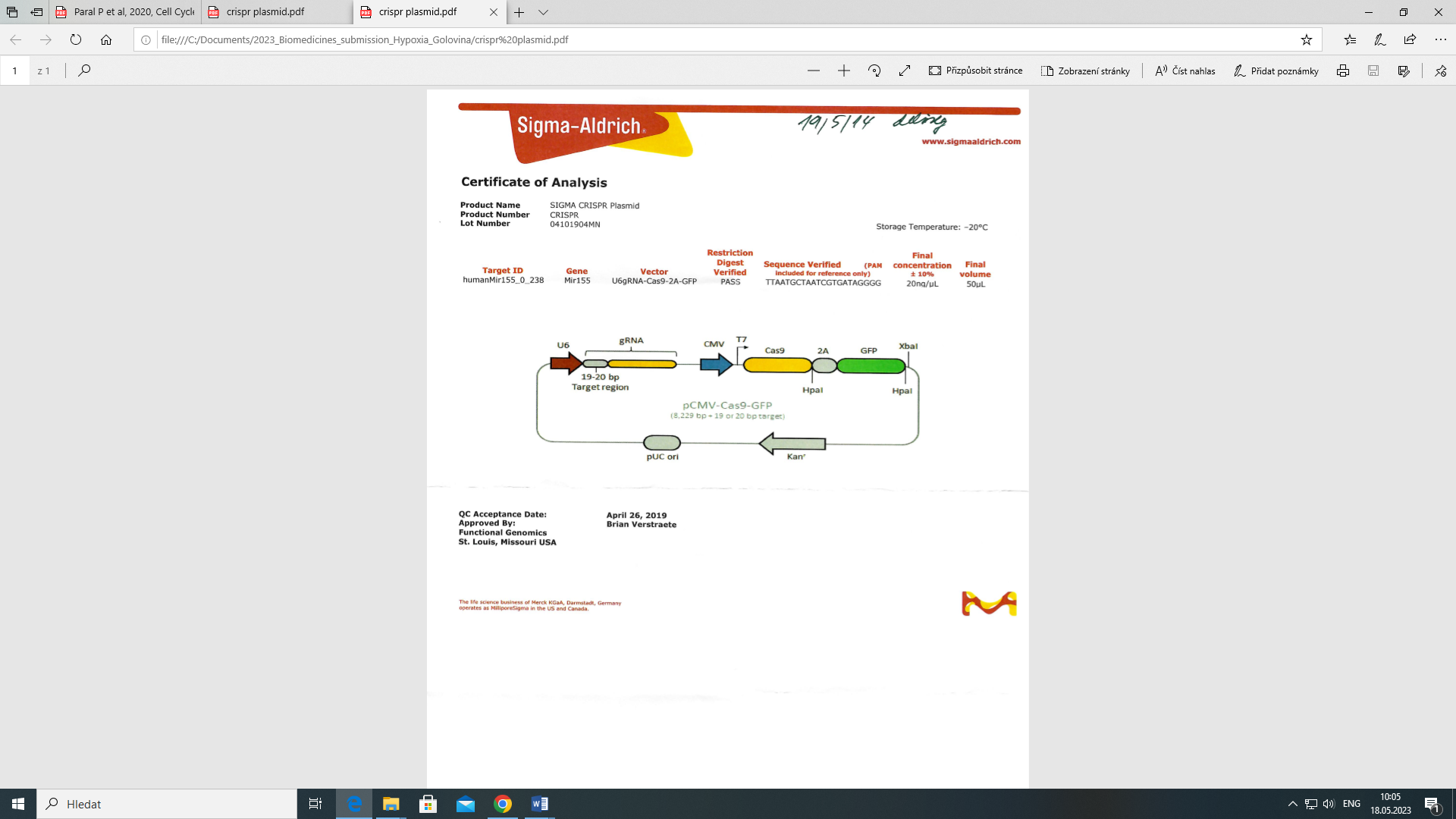

Supplement: Supplementary file 1 — Supplementary Material 1. Figure S1: Description of creation of miR-155 deficient MEC-1 cells and its validation. [file 12935_2024_3437_MOESM1_ESM.docx]
