# Supplementary material for: MiR-155 deficiency and hypoxia results in metabolism switch in the leukemic B-cells
Source: Cancer Cell Int. 2024 Jul 18;24:251. doi: 10.1186/s12935-024-03437-8 (PMC11256420; doi:10.1186/s12935-024-03437-8)
Supplement: Supplementary file 2 — Supplementary Material 2. Figure S2: Immunoblot. [file 12935_2024_3437_MOESM2_ESM.docx]

**Supplemental Figure 2**

**
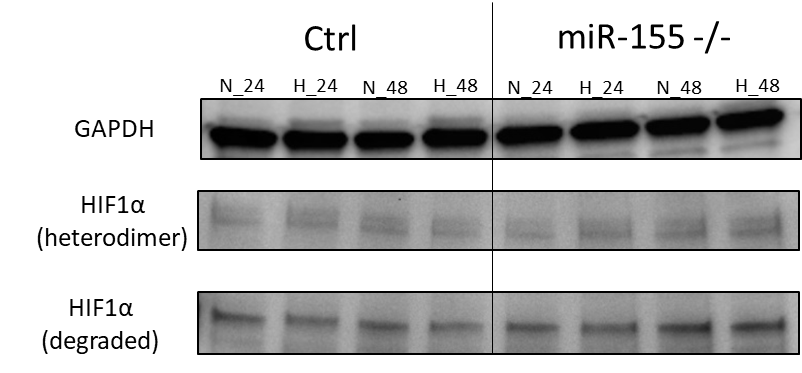
**

**Supplemental Figure 2:** Immunoblot. WB of HIF1α in MEC-1 cells after 24 and 48 hrs in 3 % O_2_. As reference gene, the protein level of GAPDH was used.
