# Supplementary material for: MiR-155 deficiency and hypoxia results in metabolism switch in the leukemic B-cells
Source: Cancer Cell Int. 2024 Jul 18;24:251. doi: 10.1186/s12935-024-03437-8 (PMC11256420; doi:10.1186/s12935-024-03437-8)
Supplement: Supplementary file 3 — Supplementary Material 3. Figure S3: Representative dot plot pictures to all flow cytometry data within manuscript. [file 12935_2024_3437_MOESM3_ESM.docx]

**Supplemental Figure 3**


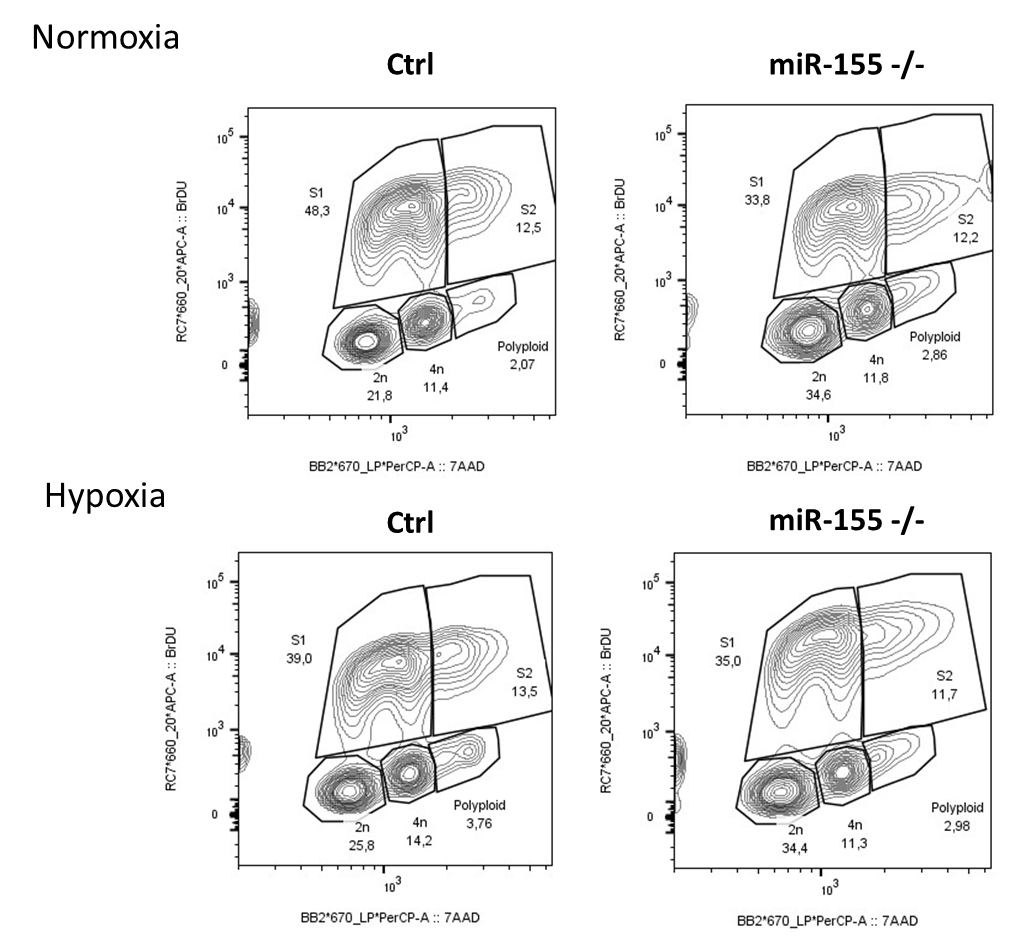


Supplemental Figures to the Figure 2D: Representative dot plots of BrdU/7AAD labeling of MEC-1 control (ctrl) and miR-155 -/- cells in normoxia vs hypoxia.


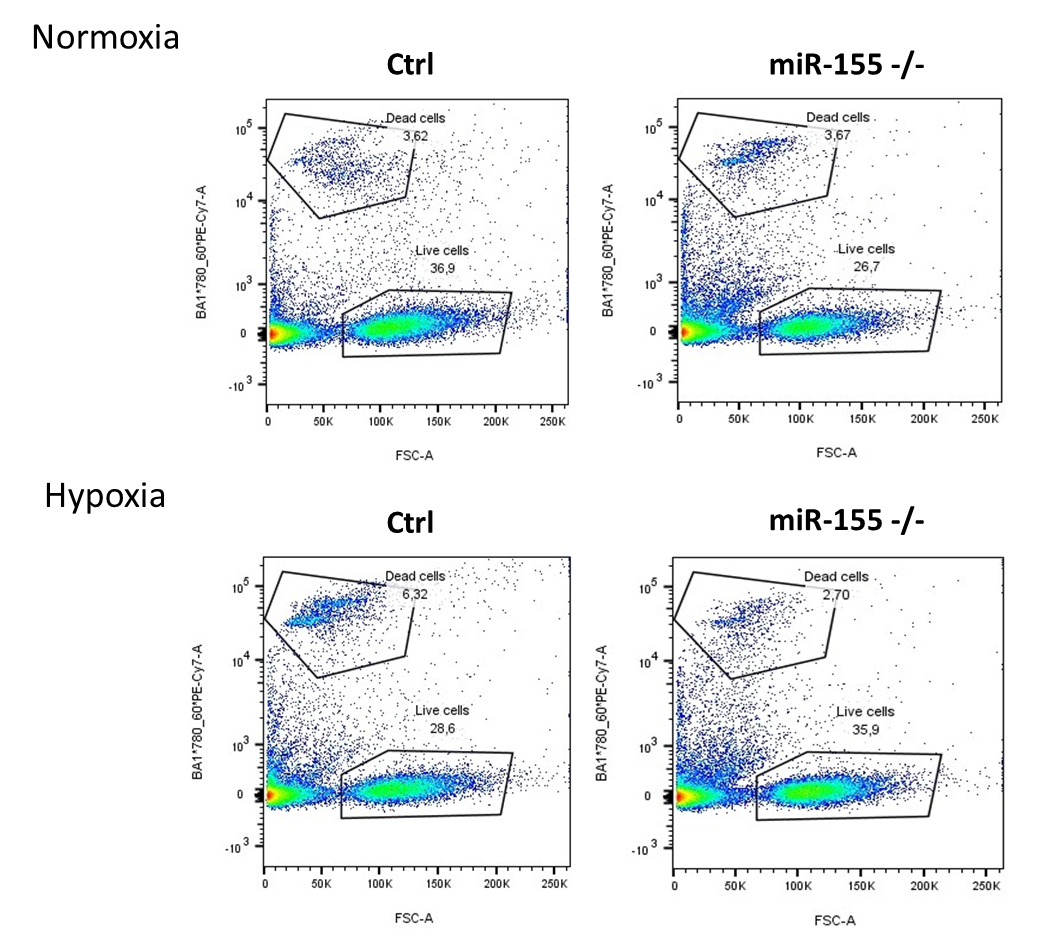


Supplemental Figure to the Figure 4: Representative dot plots of measurement of 2-DG of MEC-1 cells (ctrl and miR-155 -/-) in normoxia and hypoxia

**
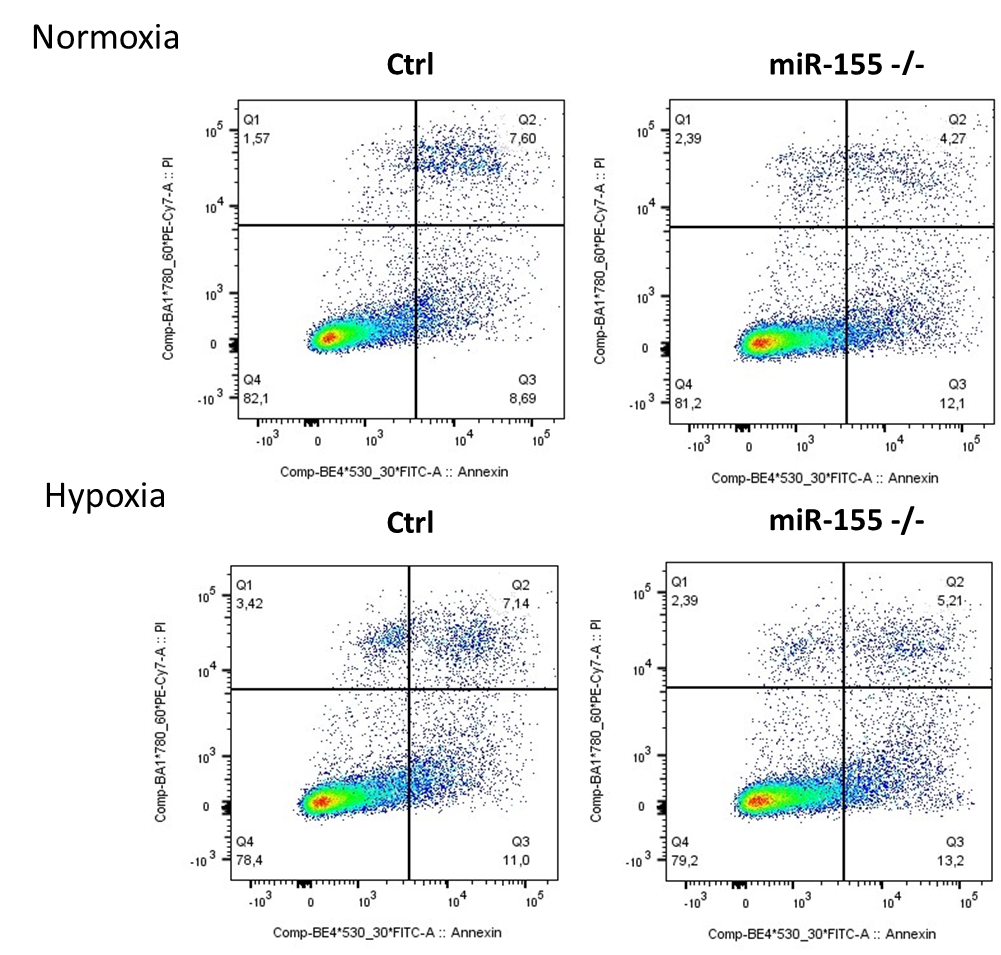
**

Supplemental Figure to the Figure 2B: Representative dot plots of measurement of AnnexinV/PI of MEC-1 cells (ctrl and miR-155 -/-) in normoxia and hypoxia

**
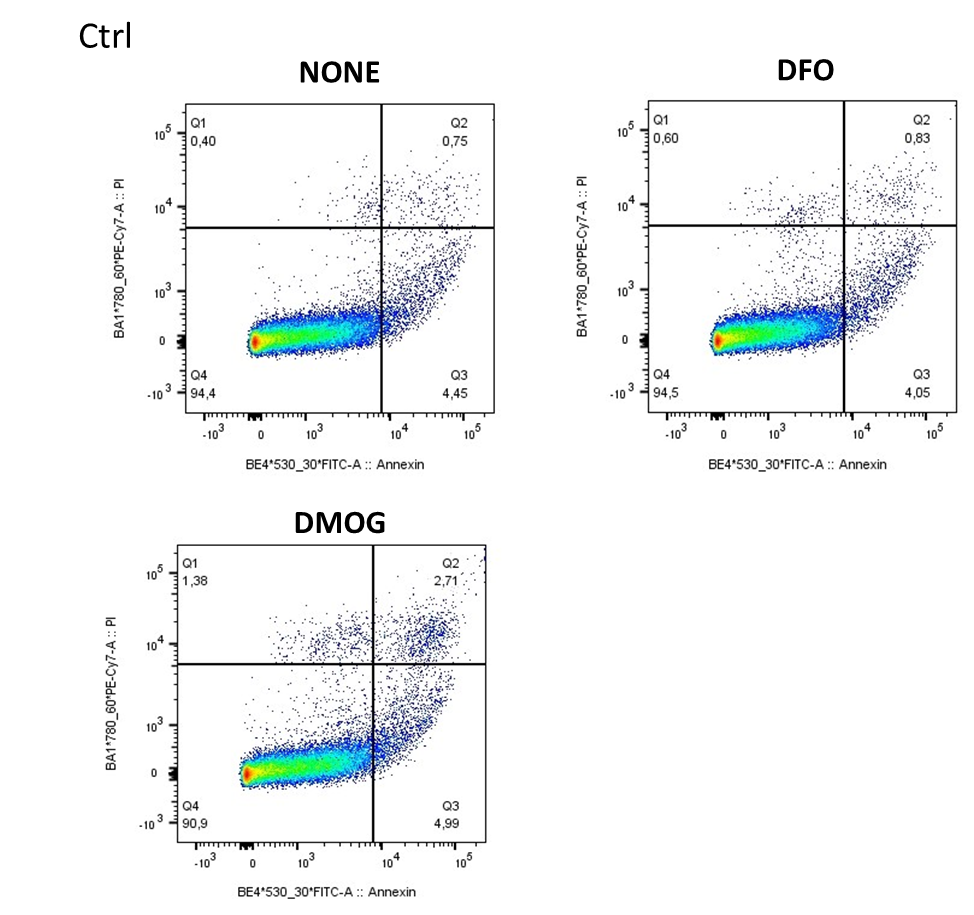
**

**
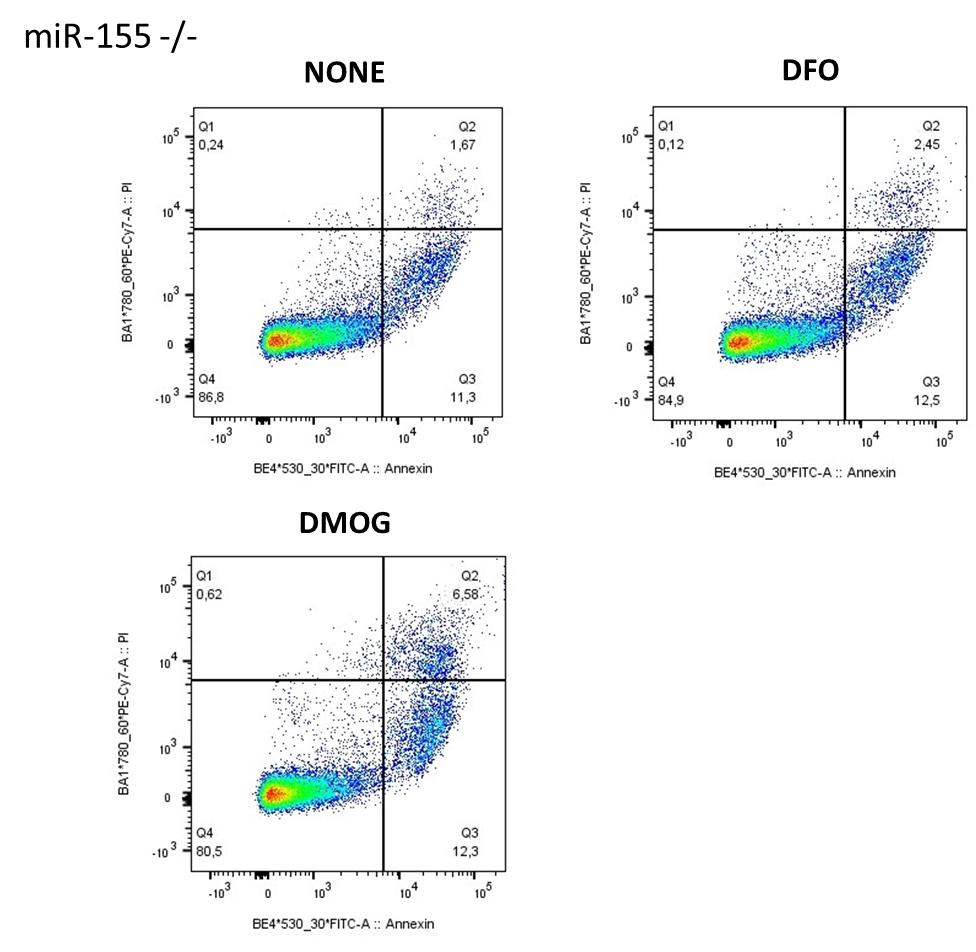
**

Supplemental Figure to the Figure 3B: Representative dot plots of measurement of AnnexinV/PI of MEC-1 cells (ctrl and miR-155 -/-) in normoxia and chemical induced hypoxia
