# Supplementary material for: MiR-155 deficiency and hypoxia results in metabolism switch in the leukemic B-cells
Source: Cancer Cell Int. 2024 Jul 18;24:251. doi: 10.1186/s12935-024-03437-8 (PMC11256420; doi:10.1186/s12935-024-03437-8)
Supplement: Supplementary file 4 — Supplementary Material 4. Tables. [file 12935_2024_3437_MOESM4_ESM.docx]

| **Day** | **Statistical significance** | |
| --- | --- | --- |
|  | **Normoxia**  **MEC-1 ctrl vs MEC-1 miR-155 -/-** | **Hypoxia**  **MEC-1 ctrl vs MEC-1 miR-155 -/-** |
| **1** | ** | * |
| **2** | *** | *** |
| **3** | *** | * |
| **4** | **** | **** |
| **5** | ** | **** |
| **6** | **** | **** |
| **7** | *** | ns |

**Supplemental Material/Tables**

**Supplemental Tables to the Figure 2**

**Tab. 1:** Statistical significance for cell growth curve (Figure 2A). Comparison of MEC-1 ctrl (control) vs MEC-1 miR-155 -/- under normoxic and hypoxic condition separately. Two-tailed unpaired t-test was used (*p<0.05; **<0.01; ***p<0.001; ****p<0.0001, ns p>0.05). Data represent the mean of 3 independent experiments.

| **Statistical significance** | | | |
| --- | --- | --- | --- |
| **Normoxia**  **MEC-1 Ctrl vs MEC-1 miR-155 -/-** | | **Hypoxia**  **MEC-1 Ctrl vs MEC-1 miR-155 -/-** | |
| ***24 h*** | ***48 h*** | ***24 h*** | ***48 h*** |
| **** | **** | **** | **** |

**Tab. 2:** Statistical significance for WST-1 assay (Figure 2B). Comparison of MEC-1 ctrl (control) vs MEC-1 miR-155 -/- under normoxic and hypoxic condition separately. Two-tailed unpaired t-test was used (*p<0.05; **<0.01; ***p<0.001; ****p<0.0001, ns p>0.05). Data represent the mean of 3 independent experiments.

|  | **Statistical significance** | | | |
| --- | --- | --- | --- | --- |
|  | **Normoxia**  **MEC-1 ctrl vs MEC-1 miR-155 -/-** | | **Hypoxia**  **MEC-1 ctrl vs MEC-1 miR-155 -/-** | |
|  | **Time period** | | | |
|  | ***24 h*** | ***48 h*** | ***24 h*** | ***48 h*** |
| **Annexin+/PI+ and PI+** | ns | ns | * | ns |
| **Annexin+/PI-** | ns | ns | ** | ns |

**Tab. 3:** Statistical significance for assessment the degree of apoptosis measured by flow cytometry (Annexin V / PI) (Figure 2C). Comparison of MEC-1 ctrl (control) vs MEC-1 miR-155 -/- under normoxic and hypoxic condition separately. Two-tailed unpaired t-test was used (*p<0.05; **<0.01; ***p<0.001; ****p<0.0001, ns p>0.05). Data represent the mean of 3 independent experiments.

|  | **Statistical significance** | | | |
| --- | --- | --- | --- | --- |
|  | **Normoxia**  **MEC-1 ctrl vs MEC-1 miR-155 -/-** | | **Hypoxia**  **MEC-1 ctrl vs MEC-1 miR-155 -/-** | |
|  | **Time period** | | | |
|  | ***24 h*** | ***48 h*** | ***24 h*** | ***48 h*** |
| **Cell cycle phase** |  | | | |
| **S phase (S1)** | **** | * | *** | ns |
| **G0/G1 phase (Diploid)** | ns | * | ns | ns |
| **G0/G1 phase (Tetraploid)** | ns | * | ns | ns |
| **G0/G1 phase (Polyploid)** | *** | ** | *** | ** |

**Tab. 4:** Statistical significance for cell cycle analysis (BrdU staining) (Figure 2D). Comparison of MEC-1 ctrl (control) vs MEC-1 miR-155 -/- under normoxic and hypoxic condition separately. Two-tailed unpaired t-test was used (*p<0.05; **<0.01; ***p<0.001; ****p<0.0001, ns p>0.05). Data represent the mean of 3 independent experiments.
